# Coaches’ insights: Determinants of athlete success, physical demands and training approaches in single-handed Olympic-class dinghy sailing

**DOI:** 10.1371/journal.pone.0322510

**Published:** 2025-06-09

**Authors:** Chelsie E. Winchcombe, Paul S. R. Goods, Martyn J. Binnie, Peter Peeling

**Affiliations:** 1 School of Human Sciences (Exercise and Sport Science), The University of Western Australia, Western Australia, Australia; 2 New South Wales Institute of Sport, Sydney Olympic Park, New South Wales, Australia; 3 Australian Sailing Team, Mosman, New South Wales, Australia; 4 Western Australian Institute of Sport, Mt Claremont, Western Australia, Australia; 5 Murdoch Applied Sports Science Laboratory, School of Allied Health (Exercise Science), Murdoch University, Perth, Western Australia, Australia; 6 Centre for Healthy Ageing, Health Futures Institute, Murdoch University, Perth, Western Australia, Australia; Universiti Malaysia Terengganu, MALAYSIA

## Abstract

Gaining insights into experienced coaches’ perceptions and understanding of performance and training can enhance knowledge to optimise athlete performance. Ten experienced International Laser Class Association dinghy (ILCA) sailing coaches with world-class and elite ILCA coaching credentials undertook semi-structured interviews to explore three key topic areas: i) **determinants of athlete success**, ii) **physical demands of competition**, and iii) **training practices and philosophies**. Hierarchical content analysis was used to establish general dimensions and higher order themes from the interview transcripts. Three general dimensions were established within the topic area of **determinants of athlete success**: i) ***sailing the boat fast***, ii) ***being a knowledgeable athlete***, and iii) ***consistent execution***. Within the topic area of **physical demands of competition** three general dimensions were also developed: i) ***hiking is the most physically demanding skill***, ii) ***environmental conditions influence athletic demands***, and iii) ***accumulation of fatigue over a regatta***. Finally, in the topic area of **training practices and philosophies** there were two general dimensions: i) ***periodisation***, and ii) ***specific training***. Overall, hiking featured across all three topic areas, highlighting its importance in ILCA sailing. Additionally, ‘feel’ and ‘keeping the joy’ were identified as higher order themes that have been under-researched in current literature. Findings suggest coaches should target consistency in both on and off-water training through ‘keeping the joy’ and sailing in a variety of conditions to improve aspects such as ‘feel’ and ‘pattern recognition’. We provide key insights into components of performance and aspects of the physical demands and training in ILCA sailing to optimise athlete performance.

## Introduction

The performance of sailors in the International Laser Class Association dinghy (ILCA) – formerly known as Laser Standard for the men’s (now ILCA7) and Laser Radial for the women’s (now ILCA6) singlehanded Olympic class dinghy – is influenced by many factors. These factors include, but are not limited to, an athlete’s technical and tactical knowledge, mental capability, physical fitness and their ability to interpret and respond to changing weather and environmental conditions [1,2]. As the most widely sailed Olympic class dinghy, ILCA sailing combines accessibility, high participation rates, and intense competition, making it an ideal setting for studying performance optimisation and training methodologies. However, despite its Olympic status and global prominence, ILCA sailing has received significantly less scientific attention than other Olympic racing sports, such as swimming, rowing, and athletics [3]. This lack of research may stem from the challenges of data collection in open-water environments, restrictions on performance-tracking technologies in competition, and historical research priorities favouring sports with more standardised conditions. As a result, there is limited understanding of the key determinants of success in ILCA sailing, the specific physical demands of competition, and the training philosophies that underpin high performance. Given the small performance margins and the complex interplay of physical and cognitive demands, coaching plays a crucial role in preparing sailors to succeed. Investigating coaches’ perspectives provides critical insights into these areas, addressing a notable gap in the literature and contributing to broader discussions in sports science, particularly in endurance, tactical, and open-environment sports.

In addition to the strategic and tactical aspects, Castagna and Brisswalter [4] suggest that the performance of ILCA sailors can be directly related to the capacity of the sailor to overcome the external forces imposed on the boat. The sailor has three controls to react to these external forces: a) changing course, b) trimming the sail, and c) altering the forces they exert on the boat [5]. Previous ILCA sailing literature has largely focused on the physical and physiological demands of “hiking”, a technique athletes use for upwind and reaching sailing to alter the forces they exert on the boat. Hiking involves the athlete leaning out over the windward side of the boat with their feet hooked under a foot strap to counteract the heeling forces of the wind on the sail, preventing the boat from tipping and ensuring it sails flat through the water [6–9]. While these studies are important for providing insights into the physical demands of hiking, they do not capture the full scope of comprehensive preparation ILCA sailors need for competition and success.

Research in other areas of ILCA sailing has explored visual search behaviour, temporal analysis, workload demands, and performance analysis [10–15]. For example, eye-tracking technology has provided insights into the visual and cognitive processes underlying decision making in sailing [13,14]. In one study on the approach and rounding of the windward mark, a number of practical applications were identified, including the importance of releasing the trimming lines close to the actual rounding and practicing against opponents [13]. Such studies are valuable for understanding how sailors process information and make real-time decisions, which is crucial for optimising performance under competitive conditions. However, these studies are limited and only provide information on isolated aspects of performance. Furthermore, the relevance of each of these aspects to training and overall performance in ILCA sailing has not been fully investigated.

To improve our understanding and enhance ILCA sailors’ performance, it is essential to further our knowledge of the competition demands and training strategies. The increased availability of global positioning systems (GPS) units has allowed greater insights into the physical and physiological aspects of regatta demands. Winchcombe et al. [15] elucidated that upwind sailing comprises approximately 60% of sailing time during races, with heart rates during upwind sailing (159 ± 11 bpm) being higher than during downwind sailing (147 ± 15 bpm, p < 0.001) and reaching (156 ± 16 bpm, p = 0.002). While this information is useful for optimising aspects of an athlete’s physical training program, it only represents data from two domestic competitions and does not investigate the training strategies that coaches use to prepare athletes for these demands.

Currently, there is a significant gap in understanding how ILCA coaches prepare athletes for competition demands. Coaching strategies are fundamental to athlete success, yet research on coaching in ILCA sailing remains scarce. Studies in other sports have demonstrated the value of qualitative research in capturing coaches’ philosophies and experiential knowledge [16–18]. For example, research on rowing coaches’ perspectives highlighted key components of performance under three themes: 1) getting the basics right, 2) targeting types of talent, and 3) the complexities of performance [19]. These insights have provided coaches, athletes and performance support staff with key information to improve rowing performance and also presented factors pertinent to junior rowers to enhance their progression in the developmental pathway [19]. Furthermore, this contribution of experiential knowledge provides researchers and practitioners with further avenues of inquiry to pursue experimental research [19].

Despite the critical role of coaching in ILCA sailing, there is a lack of scientific literature examining coaches’ perspectives on the key factors for success, their understanding of competition demands, and the strategies they use to develop winning athletes. While previous research has explored various aspects of performance, such as the physical demands of hiking and visual search behaviour, coaches’ perspectives on preparing athletes for these multifaceted challenges in ILCA sailing remain unexplored. Therefore, the aim of this study was to use a qualitative approach to explore the experiences and perceptions of ILCA sailing coaches in three main areas: 1) **determinants of athlete success**; 2) **physical demands of competition**; and 3) **training practices and philosophies**.

## Materials and methods

This cross-sectional study took a small q qualitative research approach [20] with a post-positivist perspective [21].

### Participants

Ten ILCA sailing coaches (8 male and 2 female) with at least 5 years’ experience (mean experience 11 ± 5 y, range: 5–20 y) and had previously, or were currently working with world class and elite level [22] athletes were recruited for this study. Coaches had or were currently coaching for a variety of countries (Australia, China, Denmark, Japan), with the majority (n = 9) having coached within the Australian High Performance Sport System during their career. The lead investigator (CW – female) was a doctoral student embedded within the Western Australia Institute of Sport (WAIS) with the Sailing Program and used contacts through the WAIS Sailing Program to recruit participants. Signed informed consent was obtained from all coaches, and the study was approved by the host institute’s ethics committee (2021/ET000447).

### Data generation

Prior to study commencement, an interview guide was created with lines of questioning and associated probing questions to help with the interview process. This was refined through a pilot interview with a coach working with a state institute sailing program (i.e., working with highly trained and elite level athletes) who was not part of the study. The interview guide (see supplementary material) included three general topic areas of investigation related to ILCA sailing: 1) **determinants of athlete success**; 2) **physical demands of competition**; and 3) **training practices and philosophies**. Purposive sampling was used, and participants were directly contacted (in person and via email) between the 21^st^ May 2021 and 14^th^ February 2022 to gauge their willingness to participate in the study. They were provided with a brief overview of the key questions. Only one coach declined to participate on invitation. Once a coach had agreed to participate, a meeting was arranged at a time convenient to the participant in which a verbal, one-on-one, semi-structured interview took place, either through an online meeting (n = 8) or face-to-face at either the Western Australian Institute of Sport (n = 1) or the Fremantle Yacht Club (n = 1). The lead investigator (CW) conducted all interviews, which averaged 53 ± 12 min in duration and were recorded either by laptop (HP Pavilion, HP Inc., California, USA) or phone (Samsung A23, Samsung, Seoul, South Korea).

All interviews were then transcribed verbatim using a transcription software (Otter.ai, California, USA). The lead investigator (CW) reviewed and edited (i.e., small grammatical changes) each transcript while listening to the interview recording to correct words that the software had mistaken. Each transcript was then sent back to the participant for review, to make sure their views were accurately reflected and make any amendments they deemed appropriate. The transcripts (data) were then imported into a qualitive analysis software (NVivo20, QSR International, Melbourne, Australia).

### Analysis

Hierarchical content analysis was chosen for its structured approach to identifying and organizing key themes, aligning with the study’s objectives by clearly linking individual insights to broader themes and providing actionable insights for coaching and training methodologies. Hierarchical content analysis was used to develop general dimensions and higher-order themes [23] using a predominantly inductive approach, with data being open-coded to emphasise data-based meanings from participants [24]. However, a degree of deductive analysis was employed to ensure that the open-coding contributed to data-based meanings and produced themes that were meaningful to the research questions [24]. During the transcription process, the lead investigator (CW) was emersed in the data by reading and listening to the transcripts and interviews multiple times to become familiar with the data. Codes were then created, primarily at a semantic level, where meaning in the data was coded explicitly (i.e., at the surface level of meaning) [21]. Codes were then clustered into meaningful categories that connected and fit well together, to create higher-order themes and general dimensions [23]. The codes, higher-order themes, and general dimensions were then thoroughly examined again and cross-checked against the entire dataset to ensure appropriate exploration, identification and representation of relevant information [23]. To help ensure rigour and improve the quality of analysis, a “critical friends” process was adopted with the co-authors of this paper to encourage reflexivity and explore multiple and alternate explanations and interpretations of the data and themes [25]. These structured discussions with co-authors involved critique and iterative feedback, enhancing reliability and validity by addressing biases, incorporating diverse perspectives, and ensuring findings were grounded in the data and participants’ accounts. Tables were created to display the hierarchical nature of the themes generated [23] within the three topic areas: 1) **determinants of athlete success**; 2) **physical demands of competition**; and 3) **training practices and philosophies** (see Discussion and Results section).

## Results

The results and Discussion of this paper have been combined in the section below.

## Discussion

Analysis of the interview transcripts generated the following general dimensions for each of the topic areas. For the topic area **determinants of athlete success**, codes and higher order themes clustered into three general dimensions: 1) ***sailing the boat fast***, 2) ***being a knowledgeable athlete***, and 3) ***consistent execution***. For the **physical demands of competition**, three general dimensions were also developed: 1) ***hiking is the most physically demanding skill***, 2) ***environmental conditions influence athletic demands***, and 3) ***accumulation of fatigue over a regatta***. Within **training practices and philosophies**, two general dimensions were established: 1) ***periodisation***, and 2) ***specific training***. For each general dimension, higher order themes are discussed in turn, below. Quotes are provided throughout the discussion and in Table 1 (**determinants of athlete success**), Table 2 (**physical demands of competition**), and Table 3 (**training practices and philosophies**) as context to the development of the higher order themes and general dimensions.

**Table 1:** Determinants of Athlete Success – Summary of general dimensions, higher order themes and quotes.

General dimensions	Higher order themes	Quotes
**1. *Sailing the boat fast***	a. ‘Hiking ability’	*P9: “I think primarily its speed, if you’re a bit faster. It’s something that you need to have to start thinking about getting good results. And then of course, it’s a little bit easier with Radial fleet to see that advantage compared to Full Rigs. We were focusing a lot on speed, because it’s just so much easier to be, you know, you can limit your choices and sail a little bit more conservatively when you’re faster.”*
		*P7: “The ability to hike hard is just critical. So there’s other factors at play, obviously, but I certainly think in the Full Rig fleet it’s probably only five to 10 guys that, they’ll be hiking as hard as they are at the end of the race, as to what they were at the start. It’s making these tiny little differences that just gets them a little boat here and there.”*
		*P5: “I reckon a big part of it is the ability to sustain the down force. So, you know, just the physics of that makes sense as to being able to sustain that, however, I can show you plenty of videos of people that are just hiking flat stick, and then there are other people that are hiking, not as hard, but they’ll be more dynamic, and taught, and like, physically twisting and propelling the boat, that will go faster.”*
	b. ‘Fitness’	*P1: “But first and foremost, you got to be fast. You got to get fast. So my order of getting things right, again, okay, let’s get fit. Let’s get boat handling, right. And then we’ll get fast, and then we’ll get smart on the race course. So probably in that order.”*
		*P10: “So the number one priority, if you want to become a successful Laser sailor, is keeping that boat flat, and the only way you’re going keep that boat flat, is by being extremely fit and working really, really hard.”*
	c. ‘Anthropometric characteristics’	*P4: “Probably physical attributes is a big thing. You know, it’s a very particular requirement in terms of height, weight, depending on which rig you’re using.”*
	d. ‘Feel’	*P6: “I think just in in the Laser class it might differ a lot, because it’s a low-tech class effectively. There might be a bit of a range in knowledge about aerodynamics, hydrodynamics, meteorology, and all the kind of science involved. So yeah, I suspect there’s a bit of ignorance, but it may not affect the results, because there might be people who can feel the boat really well and just do things because they know it works.”*
	e. ‘Trimming accuracy’	*P2: “But the accuracy of their steering and trimming, that’s where you will always see somebody come through, there might be somebody who can just hike. But if you don’t have the accuracy of the steering and the trimming, then they’re still not fast. So knowing what to do with that is imperative.”*
		*P6: “I think that one key skill, in terms of boat speed, which is obviously a key way to get around the course quickly, would be what we call the automatic trimming skills, which is where someone can trim and sail the boat, and then look around and make the decisions. So they’ve got the timing, the skill acquisition and technique learning, that they can have the spare attention span, to start studying the environment a lot more. It just gives more attention to their tactics and strategy and their ability to play the game a lot better.”*
**2. *Being a knowledgeable athlete***	a. ‘Ability to learn’	*P4: “There’s probably, mentally, just the ability to learn quite quickly. I’d say it’s very demanding. There’s not many, I mean, you’ve got four ropes or something, like there’s not much to play around with, comparatively to other classes. So it’s very demanding and the differences between everybody are very, very small. So you need to be picking up on the little changes, changes in pressure, changes in set up, just being able to read the fleet, so there’s that aspect of it.”*
		*P1: “I’ve coached one of the least naturally gifted guys and he’s won a world championship. And so, it’s just doggedness to learn.”*
	b. ‘Pattern recognition’	*P4: “Again, pattern recognition is probably a big thing. There’s a lot of very talented sailors, and those people who aren’t that talented, they just pick up on, oh I’ve seen this play before, in this situation I do this, in this situation I do that.”*
		*P7: “But the next one is definitely what I would call sailing intelligence, like your tactics, strategy. And a lot of that has to do with pattern recognition. But I think a lot of it can be sped up with good teamwork, you know, good conversations and feedback in the training environment.”*
	c. ‘Experience’	*P3: “Just experience in those situations, like being in the high pressure races or being in more medal races. And I think it’s having that ability to have the plays ingrained in your mind. When you get a situation, you know what the play is. You know: am I going to tack, am I gonna duck, whatever it might be. So, I think amongst the top 10, they would all have good tactical knowledge. But I guess some would have more experience of actually putting it into play than others, which is going to help when the pressure is on.”*
	d. ‘Decision making’	*P6: “And then the decision-making skills and the ability to process all the information that needs to be processed (the ability to see the wind well) and put some good decision making frameworks into practice in each race.”*
		*P8: “I think the second part would be probably around cognitive processing. So it’d be something around mental, like the mental fortitude to make decisions under pressure, because the Laser fleet goes slow, but there’s 70 of them. Yeah, so you make one mistake, and it just [claps] you know. So the ability to fight back, keep making good decisions clearly under pressure, it’s an exceptional strength, and it’s clear in the top five of both men’s and women’s.”*
	e. ‘Knowing your equipment’	*P7: “It’s an equipment based sport, and you’ve got to get the most out of it. I know Lasers are a one-design class, but certainly what I learned in the period between 2008 and 2015, there’s no such thing as a one-design class, you know. And if you think that way, you’re kind of leaving things to chance.”*
		*P8: “Understanding how to make the boat work, how to be efficient within that, you know, be successful, in that sense, they’re precursors to being able to operate at the next level, which is making good decisions strategically, then even starting rudimentary positioning or tactics.”*
**3. *Consistent Execution***	a. ‘Mental’	*P6: “Beyond that, the two other key areas on the more on the mental side, just the raw determination to excel and to push themselves hard over a long amount of time is really important.”*
		*P5: “I would then say, probably on an equal standing, maybe it’s a touch under, is the mindset or the mental aspects, and being able to execute under pressure consistently.”*
	b. ‘Physical’	*P3: “I think just the general theme of consistency because, like I said at the start, it’s just that ability to consistently perform. It’s going to come from consistently training, consistently giving a good effort in training, being consistent with your routines, your nutrition. So, I think just the general theme of consistency.”*
		*P10: “But I think, it’s the hard work, the work ethic, I think is a very big thing. Then one of the things that we’re going to work on a lot as well, is the accountability; it’s working the hardest when no one is looking.”*

**Table 2:** Physical Demands of Competition – Summary of general dimensions, higher order themes and quotes.

General Dimension	Higher Order Theme	Quotes
**1. *Hiking is the most physically demanding skill***	a. ‘Pain’	*P9: “So you need to train it, and you need to be strong, and you need to withstand the pain. So it’s definitely tough. You need to need to be well prepared to do it.”*
		*P10: “Well, even though it’s a long time ago that I sailed Laser Radials, or Lasers, I think the pain doesn’t change. You know, it’s what they say about the Laser and that will never change either, especially now with things evolving in sailing so quickly, with the foiling and stuff, is that it’s definitely the boat that I’ve never had to work so hard to go so slow, and that will never ever change.”*
	b. ‘Unnatural position’ (Biomechanics)	*P10: “I think it’s just, you’re putting your muscles and angling certain parts of your body into position that is not a natural position, at all, to be in.”*
		*P7: “I find it a very contradictory position to put your body in; it’s leaning out and contracting at the same time. I kind of see that, I said sailing intelligence before is that top-tier of things, but I see this stuff down here as body intelligence. I that sailing’s not a particularly intelligent thing to do to your body, you know.”*
	c. ‘Blood flow occlusion’	*P6: “The obstruction of the blood vessels through the muscle squeezing on them. So, some pain from that local lactate and some from the lack of blood flow I think.”*
		*P5: “So yeah, for me, and the athletes I work on, it’s definitely the occlusion that restricts that blood flow. So sure, there are techniques you can do to try and stimulate the blood flow in your legs, whilst your hiking, but I think that’s probably one of things that’s most overlooked in hiking boats, period, and then in the Laser as well. But yeah, that’s probably the hardest thing I would say.”*
	d. ‘Sustained extension’ (Strength endurance)	*P3: “I think it’s just the need to hold it for as long as you can. Like, if you’re doing a 15 minute upwind in a race, I don’t think there’s anyone in the world that would be able to hold perfect form for 15 minutes.”*
		*P2: “I think actually the ability lock in, and really have a force on the boat. Definitely the sustained extension is huge, because you’ve got to be able to hold that for 15 to 20 minutes.”*
**2. *Environmental conditions influence athletic demands***	a. ‘Strong winds’	*P1: “Because they’re all pretty tough when there’s wind and there’s been plenty of windy regattas.”*
		*P6: “The odd regatta can be 20 to 30 knots each day for 6 days; that sort of thing is just a massive hike off in terms of physically getting around the course, but also remarkable on the downwinds in the gusts. You didn’t have the arm strength to pull the sheet on, the best you could do is just maintain that level, there is no strength left to pull it on. You are so locked into the boat you can’t do anything about it. So you would just have to hope that there is a slight lull coming so you can go around the mark at the end of the run, otherwise it was just veer off or capsize.”*
	b. ‘Uncomfortable temperatures make regattas tough’ (Temperature)	*P7: “Definitely when I think about the hardest events, they usually had an element of temperature in them, that I wasn’t used to, or everyone was just really uncomfortable with.”*
		*P4: “There was a World Championships in Brazil, can’t remember how many years ago now. Where it was in the trade winds and it was 18 to 20 knots every day, all day, repeat. Quite warm, so it’s, obviously you’ve got that side as well, trying to stay hydrated and perform, and the physical demands are quite high.”*
	c. ‘Light winds are less physically demanding but more mentally demanding’	*P6: “Light winds. It’s the most mentally demanding, but the least physically demanding.”*
		*P4: “I mean, light winds. You’re just you’re sitting there, but your heart rate can spike quite a bit in light winds, like surprisingly so. There’s probably almost more tension in light winds, because it’s harder to do anything. If you’re making a mistake, and you’re caught on the wrong side, you’re probably building up pressure inside of you as well, too, because you can’t escape as easily. You know, it takes a lot longer to move from point A to point B.”*
	d. ‘Big waves increase the challenge’ (Sea-state)	*P4: “The waves are quite big, too. So created a bit more, and the waves were offset slightly to the wind. So again, if you had different techniques from tack to tack, which just made it a little bit more challenging, I guess.”*
		*P5: “We basically had 20 knots every day, three-metre swell. So it was just yeah, just brutal, brutal on the legs. Recovery was hard, [it was a] six-day event.”*
**3. *Accumulation of fatigue over a regatta***	a. ‘Backing up day after day’ (Accumulated time on-water)	*P1: “Backing up, day after day long hours on the water, plays a big part.”*
		*P6: “Speaking of windy races, windy regattas, there’s muscle damage going on from day to day, which over the course of a windy Worlds, that’s leading to a gradual deterioration in the ability to output your effort. There’s minor damage, little breaking of the bits inside the cells and cell destruction, stuff like that, which can’t quite recover quickly enough on a day to day basis.”*
	b. ‘Physical and mental fatigue’	*P2: “So if you have a light wind regatta and they’re not pushed, physically, mentally they are fatigued and physical and mental fatigue are so close that your brain hurts type of thing.”*
		*P9: “It’s the combination of having to do a tough physical workout combined with using your brain. So you’ve got to balance, you’re going to get fatigued in your head and in your body.”*
	c. ‘Multiple races on one day’	*P5: “Yeah, three-race days; they’re always the ones that separate the well prepared versus the ones that are not.”*
		*P4: “I mean, the fatigue builds up, right? You’ve got to go out, you know, to Worlds, you’re doing two races a day, which is generally a pretty good day, like a light day, relatively.”*

**Table 3:** Training Practices and Philosophies – Summary of general dimensions, higher order themes and quotes.

General dimensions	Higher order themes	Quotes
**1. *Periodisation***	a. ‘Goal setting’	*P5: “So I think it’s having that flexibility, but the ability to understand what the athlete’s goal is, and the time frame you have, when building out that program.”*
		*P10: “We all have a shared goal; we can have our individual goals, we have to have our individual goals as well, as athletes, as coaches, but we also have to have a collective goal as a squad, as a group.”*
	b. ‘Annual planning’	*P6: “So it kind of periodized the skills as the elements of the sport as a higher priority than periodizing the fitness, to some extent. There is certainly room for fitness, it kind of like comes along for the ride a lot of the time.”*
		*P5: “So the peak event, lets say the World Championships, which is generally in, I guess, Northern Hemisphere, it’s around September/ October time, then you might have a mini peak event halfway through the year as well. So you’ve got two, but everything is focused on that main peak event. So you just structure the training and a time frame back. So I like to work athletes in six-week training blocks, generally, the sixth week is a recovery or deload week. So I like to work back in six week training blocks, and we’ll structure based on the needs and/or other events that they have from there.”*
	c. ‘Volume of training’	*P3: “So, I guess I’ll talk for the top sailors, like state level and national level sailors. Typically they are doing five day weeks when they are in training, with between two to three hours on the water per session. So 10 to 15 hours on water… probably more like 12- 15 hours on water.”*
		*P6: “I guess as a big picture I’d think globally, in terms of the volume of hours spent, like 60-65% should be on the water and 40% or so off the water, in a combination of endurance, which is typically cycling, to strength. For some people it might be half and half.”*
	d. ‘Mirroring regatta demands in training’	*P2: “I think understanding the regatta load and then trying to match that or get close to it in training is important. And so I think in that lead up, you need to have some really good strong hours of training blocks, and then build it into regatta competitions and then go back into your training, and really consistent with things like gym and bike to keep that fitness component but also reach those TSS scores that you need each week to match the regatta load.”*
		*P3: “That would even be less than a regatta load, so I guess that’s something we’re actually looking at now is, do we increase that to 20 hours, a month before? Or however long before a regatta, to replicate or even exceed the regatta load. Certainly, when a regatta comes it’s more natural.”*
	e. ‘Tapering’	*P1: “So understanding the peak loading periods, and then the tapering off, the correct tapering off. So basically going into the regatta, the sailor has to be fresh, absolutely, that’s without question.”*
		*P2: “And then, you know, it might be sort of averaging three hours a session and then backing off as you come into worlds, you might go down to two, the volume reduces, but the intensity stays high. But more and more starting and racing starts, become part of the sessions.”*
	f. ‘Keeping the joy’ (Motivation)	*P10: “But creating that environment where we’re having fun together, and everyone’s motivated to keep working hard, and pushing them to the limits, but not pushing them over the limits.”*
		*P7: “And if you genuinely had a joyful experience, it’s all been worthwhile. You can see the value in whatever happens up to that moment anyway, it’s going to put you in a much better place, mentally, to perform, because you know that whatever happens, it’s been a good trip.”*
**2. *Specific Training***	a. ‘Hiking training’	*P1: “The best form of hiking fitness comes from hiking itself. You can have all these other things and, you know, you can build the capillaries in your legs by doing lots of cycling and all that. The motions different, and yeah, you build the capillaries to transport blood and oxygen through your system. Important. However, do a lot of hiking, and you’ll be able to hike, and yeah, lots of squats. So it’s absolutely imperative there is a good gym program. Absolutely, important. But without getting the big miles in hiking as well, it’s not going to compliment anything.”*
		*P6: “I think there’s a big role for specificity of training and doing some hiking bench work, supplementary if you haven’t had a lot of windy days, you know if there is a windy regatta coming up, then, you know, hiking bench work is really important to keep the legs a bit mushy.”*
	b. ‘Boat speed’	*P9: “Probably 80%. So a big part of our training is speed.”*
		*P6: “Before that, we’ll go through a cycle a couple of times where we work on boat speed for a month or so, fitness with that, not worrying about results or peaking, and then go into a mode where we work on racing a little bit more, maybe in smaller regattas, like the domestic regattas, and then cut that off again, and go back to working on boat speed for a period again.”*
	c. ‘Off-water training’	*P8: “I think the off-water is just as important, especially earlier in the season; the off-water stuff is actually the higher weighting.”*
		*P9: “We have a physical preparation coach, so I’m pretty sure they do training five days a week. It’s a mix of strength and cardio.”*
	d. ‘Training mental capabilities’	*P9: “It’s hard to train. Because, you know, you can maybe change it, in experiencing the situation, but it’s very hard to create the same kind of load, and tension, and pressure.”*
		*P10: “It’s more challenging for us, I think, as coaches as well, to get our sailors to reach that mind frame, or that mindset, that’s necessary for them as an individual to perform, than getting them to that hiking fitness level.”*

### Topic Area 1: Determinants of athlete success

Coach definitions of athlete success in ILCA sailing ranged from explicitly winning an Olympic gold medal and/or a World Championship, to helping athletes accomplish their goals and improve their performance. As many of the coaches had experience across the developmental pathway, they also highlighted that the level of the athlete/s they were coaching is an important factor when considering success.

#### General Dimension: *Sailing the boat fast.*

Five higher order themes clustered together to establish the general dimension of ***sailing the boat fast***: ‘hiking ability’, ‘fitness’, ‘anthropometric characteristics’, ‘feel’, and ‘trimming accuracy’ (Table 1). Each of the higher order themes represent key aspects that coaches discussed as being crucial to boat speed (i.e., sailing the boat fast).

Hiking is an important aspect in ILCA sailing, especially when winds exceed ~8 knots [11,26]. ‘Hiking ability’ was raised by coaches as key to sailing a boat fast upwind. When referring to ‘hiking ability’, coaches referenced both the ability to hike at full extension, and sustain that extension:

*“The ability to, I guess, move your body around appropriately is important for speed. But you’ve got to be able to get the extension, and be able to hold that extension.” [P1]*

Pan and Sun’s study [12] of the 2019−2020 Hempel World Cup Series and Tokyo Olympic Games reported a moderate to high correlation between first upwind mark position of sailors and their final race ranking (r ranging 0.762–0.851, p < 0.01) across all three wind conditions (<8knots, 8−12knots and >12knots). This result indicates the importance of hiking ability as well as strategical, tactical, and starting ability to regatta performance. Additionally, the correlation between hiking endurance (i.e., sustained extension on a hiking bench) and performance in major regattas has been shown to be inversely high (r = −0.82; p < 0.05), with longer hiking duration on the hiking bench associated with a better finishing position in racing [27].

The higher order theme of ‘fitness’ was often directly related to the athlete’s ‘hiking ability’ by coaches. Previous investigations have indicated a range of correlations when examining the relationship between aerobic fitness and regatta performance. For example, Legg et al. [2] studied New Zealand sailors preparing for the 1996 Olympics and found that correlations between regatta results and 2500m rowing score for six ILCA7 sailors were r=0.22 at Sail Auckland and r=0.70 at the Laser Nationals in moderate winds, and r=0.62 at the Olympic Trials in light winds. Although this study had a small number of participants, these outcomes highlight the importance of other factors such as skill and tactical ability to overall racing performance in an ILCA. Additionally, Tan et al. [26] identified that of 13 physical parameters (e.g., vertical jump, cycling time to exhaustion, three repetition max strength), only body mass (r=-0.69, p<0.01), and maximal hiking moment over 180 s (r_s_=-0.62, p<0.01) were associated with better racing results in male ILCA sailors during a National Inter-School Laser Championship in Singapore. This concurs with the coaches’ views that fitness, in terms of ***sailing the boat fast,*** and performance are strongly related and associated to hiking ability. Future studies with larger sample sizes and a greater number of competitions in varying wind conditions may better elucidate whether more traditional markers of aerobic fitness (e.g., maximal oxygen uptake etc.) relate to competition performance.

All coaches agreed on the importance of *“being the right size” [P6, P7, P8]*, with ‘anthropometric characteristics’ identified as a higher order theme attributing to the general dimension of sailing the boat fast:

*“If I broke it down technically, from the performance of Laser sailing, you’ve got start with your body. I think you’ve got to be the right size, you know, we’ve got to be in the range of the right body weight, and the right, just basic levers and functionality.” [P7]*

This is particularly important to be able to create a righting moment when hiking to counteract the force of the wind in the sail [6,28,29]. Recent research featuring athletes in the ILCA7 class reports the anthropometric characteristics of 11 elite males (age: 23.2 ± 3.4 y) as 182.5 ± 5 cm and 82.6 ± 2.3 kg [15]. Additionally, a study of Chinese sailors competing at the 2020 Chinese National Championship reported the anthropometrics of 27 ILCA6 sailors (age: 20.5 ± 4.1 y), with a height of 171.4 ± 3.6 cm and body mass of 60.8 ± 4.3 kg [30]. Coaches also emphasised that venue-specific conditions, such as wind strength and sea state etc., significantly influence what would be considered as optimal anthropometric characteristics. For instance, coaches explained that lighter sailors may excel in lighter winds due to reduced drag, while heavier sailors benefit from a greater righting moment in stronger winds, enabling them to maintain a flatter boat and increased upwind speed. Additionally, coaches mentioned equipment and rule changes over time have driven shifts in anthropometric requirements, as seen in the rise in ILCA7 sailors’ body mass from ~75–79 kg in the 1990s and 2000s [7,31,32] to recent years.

Additionally, the concept of ‘feel’, representing a sailor’s capacity to harmonize boat movement with wind and wave conditions, is frequently associated with innate talent. Several coaches have identified ‘feel’ as a crucial factor influencing an athlete’s capability to optimise boat speed under varying conditions:

*“So, I would say more upwind and downwind we focus a lot. And, you know, on downwinds it’s also VMG (velocity made good) and all that feel and knowledge, so you’re not going fast and in the wrong direction.” [P9]*

To the authors’ knowledge, no literature in sailing has described this phenomenon. However, a recent study that explored expert rowing coaches’ perspectives on performance indicators, also highlighted ‘boat feel’ as an intrinsic trait or innate quality of importance and association with more technically competent athletes [19]. Similarly, in swimming, having good “feel for the water” (which can also be referred to as smoothness) is a factor that can distinguish between elite and non-elite swimmers, and although it is a subjective expression, it is an important factor to improve within a training program [33].

Finally, ‘trimming accuracy’ and automatic trimming skills were noted as imperative to sailing the boat fast. While previous research in ILCA sailing has not specifically explored the technical ability of trimming (i.e., adjustment of the sail), one study has explored the physical forces associated with mainsheet trimming [34]. Preliminary investigations into the force of trimming the mainsheet when upwind sailing indicated that the average mainsheet force for a club sailor increased, as expected, when the wind strength increased from the 5-10 knot (78 N) range to 10-15 knots (81 N) [34]. However, the opposite trend was evident for an elite sailor, where the average mainsheet force decreased in stronger winds (5-10 knots: 97 N, 10-15 knots: 81 N) [34]. These results were attributed to a potential difference in skill, where the elite sailor made more frequent adjustments to the mainsail under higher wind conditions, possibly leading to a lower overall tension in the mainsheet. Although this observation is limited by factors such as uncontrolled sail settings, individual body weight differences, and wave variations, it still provides valuable insights into the relationship between skill and trimming technique [34]. These findings align with broader research using Computational Fluid Dynamics (CFD) and Velocity Prediction Programs (VPP), which indicate that maintaining optimal sail shape and depowering strategies are critical for performance [35–37]. Ma et al. [36] further demonstrated how adjustments in sailing angle, attack angle, pitch angle and camber ratio influence sail aerodynamics, providing insights into trimming during different race stages (e.g., start, upwind, downwind leg etc.). While these studies primarily model ideal trimming strategies, the current findings – both from real-world force measurements and from the coaches’ observations in this study – provide empirical support that precise sail trim is a key determinant of performance. Therefore, the collective evidence of these investigations reinforce the importance of skill and technical training for ILCA sailors.

Overall, these higher order themes within the ***sailing the boat fast*** general dimension are key aspects that primarily align with the physical characteristics and technical abilities of the athlete. Coaches highlighted that ‘hiking ability’ is a key element in the success of an ILCA sailor, which is congruent with the current literature in this space. ‘Fitness’ was often related by coaches to hiking ability, underscoring the importance of the specificity of fitness for sailing. Additionally, the importance of ‘feel’ across different conditions (wind, sea-state, current etc.) is a factor that coaches, support staff, and researchers could investigate to ensure athletes are exposed to a wide range of conditions during training to optimise their learning opportunities. Furthermore, ‘anthropometric characteristics’ could help provide guidelines for talent identification, as well as performance support practitioners working with athletes to change body composition. Finally, the importance of ‘trimming accuracy’ to ***sailing the boat fast*** should be considered in a coach’s technical framework and feedback during on-water training sessions. In summary, encompassing tailored fitness programs, diverse on-water experiences, targeted talent identification, and comprehensive technical coaching are crucial to develop athletes capable of ***sailing the boat fast*** and excelling in competitive sailing environments.

#### General Dimension: *Being a knowledgeable athlete.*

Five higher order themes grouped together to develop the general dimension of ***being a knowledgeable athlete***: ‘ability to learn’, ‘pattern recognition’, ‘decision making’, ‘experience’ and ‘knowing your equipment’ (Table 1). For a long time, physiological and biomechanical factors have been heavily researched and emphasised in differentiating levels of sports performance, however, since the 1980s, the importance of the cognitive system in sport expertise has garnered more attention [38,39].

The first higher order theme was ‘ability to learn’. This was highlighted by several coaches, with one describing ability as:

*“Ability is probably the ability to learn, whereas if it was our natural ability, everyone can see what that is. But ability also comes under the guise of being able to learn and change.” [P1]*

It has been reported that professional athletes have extraordinary skills for rapidly learning unpredictable, complex, and dynamic visual scenes that are void of any specific context [40]. This rapid learning in complex and unpredictable dynamic contexts is highlighted as a critical component for elite performance [40]. In the coaching literature, creating an environment focussed on learning has also been highlighted as a key factor in the coaching process [41].

The higher order themes of ‘pattern recognition’, ‘decision making’, and ‘experience’ are closely related but are also distinct. ‘Pattern recognition’ is the perceptual-cognitive skill related to superior recall, detection and recognition of patterns of play, more efficient and appropriate visual search behaviours, and better anticipation of likely events [39]. Many coaches highlighted the importance of good ‘pattern recognition’ in both tactical and strategical aspects of performance:

*“what I would call sailing intelligence, like your tactics, strategy. And a lot of that has to do with pattern recognition.” [P7]*

‘Pattern recognition’ allows sailors to process environmental cues, such as wind shifts, wave formations and fleet movements, more effectively, leading to faster and more accurate decision making [42]. This has been well documented in other elite level sports, such as hockey and football, where it has been shown that pattern recognition skill is an important component of performance and can distinguish between greater and lesser skilled players [43]. However, the only research in sailing to date has focussed on computer simulations [42] and junior athletes [44], highlighting a clear opportunity for further investigation into how elite ILCA sailors develop and apply pattern recognition skills in real-world conditions.

Additionally, ‘experience’ was emphasised as a higher order theme related to partaking in regattas, especially high-calibre international regattas, and the ‘experience’ and learnings of being in more high-pressure situations:

*“Watching the tracking of the Olympic racing right now and seeing some of the sailors coming from in the teens to be in the top few is what really makes them successful. It’s having that ability to just come back to it, to keep the score. That comes from experience.” [P3]*

In support of this idea, both age and sailing experience have been reported as key factors in the performance success of Olympic-class sailors [29,30]. Moreover, this aligns with Henriksen et al. [45] who highlighted the importance of gaining experience at high level international competitions as part of the talent development process in another Olympic sailing class (49er).

Furthermore, ‘decision making’ can be defined under an ecological dynamics perspective as a perception-action skill which is focused on the performer-environment system [46]. Coaches mentioned both having the cognitive ability to process the necessary information for decision making, and the ability to make good decisions under pressure, as key sailing success factors. ‘Decision making’ in sailing has been researched through computer simulations [42] and visual search and movement behaviours [13]. Pluijms et al. [13] found that better performances during a windward mark rounding were related to releasing the trimming lines closer to rounding the mark and gazing to the tangent point (i.e., single stationary point referred to as the apex) during the actual mark rounding. These findings suggest that skilled sailors engage in highly specific visual and movement behaviours that enhance their decision making efficiency. However, these findings are limited to an isolated aspect of sailing performance (windward mark rounding), whereas our study further extends the importance of both cognitive processing and the ability to make decisions under pressure as key determinants of success. Our coaches’ insights highlight the need for training strategies that develop an athlete’s ability to process complex, rapidly changing environmental cues and execute optimal decisions in high stress competitive scenarios.

Finally, the importance of ‘knowing your equipment’ was also highlighted by coaches. Even though the ILCA is a one-design class, coaches made note of still having intimate knowledge of your own boat to be able to sail it optimally, due to small differences between boats:

*“Especially if you go to the Olympics, and you’re getting your boat given to you, you’ve got to make sure that you don’t have a lemon, and you got to know what a lemon is, you know, because you’re probably not gonna get the best boat you’ve ever had, but you got to make sure it’s not a bad one. And if it’s slightly different, there’s different techniques you can change to deal with it, but if you don’t understand that, you’ll get a silver medal, but you probably won’t get a gold, or you’ll drastically reduce the chance.*” *[P7]*

Overall, these five higher order themes relate to an athlete’s cognitive capability which is a known key factor in elite athlete success [39,47]. Coaches and performance support practitioners should carefully consider and plan the integration of the aforementioned themes into the training program, including utilising regattas to gain experience and spending time to work on having a deep understanding of equipment. Previous research has shown that an athlete’s decision making skills can be enhanced by integrating mental skills training and simulated pressure situations into their training regimes [39]. Additionally, incorporating structured feedback such as debriefing and leveraging competitive experiences as learning opportunities are also crucial for developing the mental aspects critical for success in elite sports [48].

#### General Dimension: *Consistent execution.*

Within the general dimension of ***consistent execution***, the higher-order themes were distinct in ‘physical’ and ‘mental’ elements (Table 1).

When referring to ***consistent execution*** from a ‘physical’ sense, coaches referred to the consistency of training and work ethic. This is synonymous with the literature, in which training consistency over many years is cited as a key factor in becoming a successful athlete [41,49].

From the ‘mental’ aspect, coaches spoke of the mindset to consistently execute under pressure, the determination to push themselves to excel, and the ability to self-regulate, as important factors in consistently executing elite performances:

*“There’s the certain amount of mental consistency in how they go about putting decision making into action and some people don’t have that self-regulation sorted out, to have that level headedness for the whole time.” [P6]*

Previous research shows that world class athletes (n = 10, all with either Olympic gold medals and/or world title/championship gold medals in individual or team sports) all referred to their psychological competencies, rather than physical attributes, when asked “to what do you attribute to your success?” [47]. This, combined with our findings, highlights that both athletes and coaches appreciate the importance of mental competencies, such as strong and enduring self-regulation, strong intrinsic motivation, work ethic, preparation and hunger for self-challenge, in determining athletic success [47].

The over-arching general dimension of ***consistent execution*** relates to both the mental capabilities highlighted in the general dimension of ***being a knowledgeable athlete***, and the physical characteristics and technical abilities highlighted in ***sailing the boat fast***, where consistent training and practice are needed to improve these factors. Practically, this could mean ensuring that an athlete is consistently training and being exposed to competition events to ensure they develop their physical, technical, tactical, strategical and mental capabilities to perform at their best. Further information and considerations on some of these elements are covered in the topic areas **physical demands of competition** and **training approaches and philosophies** below.

### Topic area 2: Physical demands of competition

To help best inform an athlete’s physical preparation training program, it is imperative to have a good understanding of the physical demands of competition. This information, along with the different aspects highlighted as being important to athlete success in the section above, can help coaches and performance support practitioners prioritise key areas to focus on within the training programme.

#### General Dimension: *Hiking is the most physically demanding skill.*

All coaches expressed that ***hiking is the most physically demanding skill*** of ILCA sailing. Four higher order themes were established to give an understanding into why coaches thought hiking was so demanding: ‘pain’, ‘unnatural position’, ‘blood flow occlusion’, and ‘sustained extension’ (Table 2).

‘Pain’ and the ‘unnatural position’ of hiking were both referenced by many of the coaches as contributing to the demanding nature:

“*just an unnatural position for your body as a human being… something that your brain has to get used to, and you just have to get through that pain. You have to get through that pain, and then you go from there.” [P10]*

Hiking has been previously cited as the most demanding aspect of ILCA sailing, with heart rates being significantly higher when sailing upwind (i.e., when hiking) than when reaching or downwind sailing [7,15,50]. Additionally, poor hiking technique and inadequate strength in the hamstrings, quadriceps and trunk have been cited to increase the risk of injuries of the lumbar and thoracic spine and knee, which are most common injuries in Olympic class sailing [51].

When discussing the mechanisms of why hiking is so painful and physically demanding, ‘blood flow occlusion’ was referred to by several coaches. Vogiatzis et al. [52] were able to confirm that there is a reduced oxygen availability in the quadriceps muscle during hiking, due to the restricted muscle blood flow caused by the isometric contraction.

Finally, the importance of ‘sustained extension’ (i.e., holding the hiking position) with proper form (i.e., technique) throughout an upwind leg, was highlighted as a significant factor in the physically demanding nature of hiking. This has previously been investigated, where 12 Europe (similar to an ILCA6) dinghy sailors (seven males, five females, 18 ± 2 years, top 20 of Danish national rankings) showed a ~ 30% drop in hiking strap force between the first and last minute of a 10-min upwind sailing period [53]. This highlights the significant fatigue that occurs during a ‘sustained extension’, emphasising the physically demanding nature of the task.

As ‘hiking is the most physically demanding skill’ of ILCA sailing, it is critically important for coaches and performance support staff to pay close attention to technique and the sailor’s ability to maintain this technique throughout an entire upwind leg. This focus is essential for reducing the risk of injury and optimising upwind boat speed. More details on ‘hiking training’ are covered below in the topic area of **training practices and philosophies,** general dimension: ***specific training***.

#### General Dimension: *Environmental conditions influence athletic demands.*

Four higher order themes clustered together to develop the general dimension of ***environmental conditions influence athletic demands***: ‘strong winds’, ‘uncomfortable temperatures make regattas tough’, ‘light winds are less physically demanding but more mentally demanding’, and ‘big waves increase the challenge’ (Table 2).

‘Strong winds’ were referred to by most coaches as the factor with the biggest impact on the physical demands. Coaches primarily used their own experiences of sailing in windy conditions to recount the impact that the wind had on the physical demands, and how tough regatta/racing was. A previous investigation of two regatta competitions found that for every one knot increase in wind speed, heart rates of sailors increased three beats per min when sailing upwind, downwind and reaching [15]. Additionally, the fractional utilisation of maximum oxygen uptake, percentage of maximum heart rate, and post-test blood lactate values have been shown to be strongly correlated with wind speed during a 10 min on-water upwind sailing test [54]. Moreover, it has been previously established that during a 30-minute upwind test, a linear relationship exists between wind intensity and energy demand (r = 0.85, p < 0.01) [4].

The higher order theme of ‘uncomfortable temperatures make regattas tough’ was developed to recognise both ends of the temperature spectrum. Without careful mitigation strategies, exercise performance has been shown to be reduced in both hot and cold climates [55,56]. Knowledge of these factors, and expected forecasts at regatta locations, can help in the preparation of athletes by implementing appropriate mitigation strategies (i.e., pre-cooling, acclimation etc.). Previous research has shown that heat strain was reduced in elite ILCA sailors following a five-day heat acclimation protocol, with most thermoregulatory adaptations retained for two weeks afterwards [57]. Implementing a brief heat acclimation protocol and periodic re-acclimation sessions has shown to be beneficial in mitigating performance decrements induced by elevated temperatures when athletes are not acclimated [58]. This is especially relevant for ILCA sailors, as their regatta schedules frequently involve transitioning from a cold or cool winter in one hemisphere to a hot summer in the other hemisphere.

The higher order theme of ‘light winds are less physically demanding but more mentally demanding’, was developed from coaches emphasising the influence of wind conditions on the interplay between mental and physical demands during competition. This included the added pressure of mistakes costing more in light wind conditions (expanded on in Table 2). This perspective contradicts the findings of Gonzales et al [59], who reported that young Optimist sailors exhibited poorer cognitive performance after sailing in strong winds. However, their results also showed that higher performing sailors (based on results at regional and national regattas) maintained cognitive performance across all conditions (no wind, light wind: 2–6 knots, strong wind: 11–15 knots) suggesting that the skill level of the athlete plays a role [59]. Further research, particularly in ILCA sailing, is needed to explore this interplay between mental and physical demands during both training and competition. The relationship between mental and physical fatigue is further explored in the general dimension: ***accumulation of fatigue over a regatta***.

Coaches also stated that ‘big waves increase the challenge’, and therefore, the physical demands during competition. Prior work mentions the increased physical demands encountered due to waves via the quote “the impact of environmental factors such as wave motion, wind fluctuations and body movements were responsible for a significant amount of the forces produced when sailing” [34] (p. 84). However, there has been no other investigations into the impact of sea-state on the physical demands of ILCA sailing.

Together, these higher-order themes emphasise the importance of exposure to various conditions, accurate forecasting of competitive environments, and the implementation of strategies like heat acclimation and off-water training preparation to adequately prepare athletes for diverse and challenging sailing conditions. Additionally, further research is required to understand the influence of sea-state on the demands and the relationship between mental and physical demands across wind conditions.

#### General Dimension: *Accumulation of fatigue over a regatta.*

The general dimension of ***accumulation of fatigue over a regatta*** was developed through the clustering of three higher order themes: ‘backing up day after day’, ‘physical and mental fatigue’ and ‘multiple races on one day’ (Table 2).

Coaches observed that consecutive days of racing (i.e., ‘backing up day after day’), without sufficient recovery, contributed to the physical challenges of competition because the fatigue experienced couldn’t be fully recuperated before the following racing day. Most regattas are held over five to six consecutive days (except for the Olympic Games and some World Championships, where there are reserve/rest days) and usually consist of two 45–60 min races and ~3–4 h on-water per day. This may be a significant factor in the accumulation of fatigue over a regatta, especially if the physical demands of daily racing exceed those experienced in training. However, the direct link between daily and weekly training load and ILCA regatta demands has not yet been investigated. The impact of back-to-back racing days and accumulated time on water on fatigue has previously been reported, with a more demanding regatta schedule (i.e., greater number of races and time on-water) resulting in an increase in subjective fatigue [15].

Furthermore, fatigue was also referenced in terms of both ‘physical and mental fatigue’ with coaches elucidating the closeness and intertwined nature of both mental and physical fatigue:

*“It’s the combination of having to do a tough physical workout combined with using your brain. So you’ve got to balance, you’re going to get fatigued in your head and in your body. So the more, the better you train, then it’s going to be easier to cope.” [P9]*

Mental fatigue can negatively influence an athlete’s performance, with research revealing reduced performance in tasks involving a high degree of technical skill and endurance-based activities [60]. However, the best way to monitor and manage mental fatigue in applied scenarios, such as sporting competitions, warrants further investigation [60].

Additionally, in close relation to ‘backing up day after day’, the impact of ‘multiple races on one day’ was also highlighted by some coaches as a major contributor to the accumulation of fatigue across a regatta. Generally, racing is scheduled for two races per day; however, with weather delays and abandoned racing there can often be three races per day for multiple days within a regatta. This was demonstrated in a recent study which observed greater increases in ILCA sailors’ perceptual fatigue when there was multiple three race days over the course of a regatta, due to un-sailable conditions on one of the regatta days [15].

Therefore, with the ***accumulation of fatigue over a regatta*** it becomes critical to ensure athletes are well prepared for the demands of competition. More details on ‘volume of training’ and ‘mirroring regatta demands in training’ are covered below in the topic area of **training practices and philosophies,** general dimension: ***periodisation.*** Additionally, these higher order themes highlight the importance of incorporating and utilising both physiological and psychological recovery strategies during regattas to minimise the influence of physical and mental fatigue on performance. Although there is limited research in managing physical and mental fatigue in Olympic sailing, strategies such as mindfulness have been shown to be effective in attenuating mental fatigue in other sports, especially during heavy competition periods [61,62]. Additionally, strategies such as cold water immersion have been shown to mediate perceptions of fatigue and leg soreness [63]. However, to optimise performance during competition and training, coaches and the performance support team should ensure athletes have opportunities to practice individualised strategies for managing both mental and physical fatigue during training. This proactive approach allows athletes to integrate and refine effective fatigue management techniques into their routine, rather than introducing unfamiliar interventions during regattas.

### Topic area 3: Training practices and philosophies

Within the topic area of training practices and philosophies, coaches revealed key aspects of how they prepared their athletes for the demands of competition and how they structured their training programme.

#### General dimension: *Periodisation.*

The concept of periodisation is the cyclical method of managing training variables to increase the potential for achieving specific performance goals [64]. To develop the general dimension of ***periodisation***, six key higher order themes clustered together (Table 3): ‘goal setting’, ‘annual planning’, ‘volume of training’, ‘mirroring regatta demands in training’, ‘tapering’, and ‘keeping the joy’.

The first higher-order theme, ‘goal setting’, is a key feature of periodisation and the holistic coaching process, aimed at facilitating change and improving performance [65]. Coaches focused on both individual and squad ‘goal setting’. In close relation to ‘goal setting’ was ‘annual planning’, which is the process of organising the year into different phases of training to prepare for specific competitions [66]. Coaches stated the importance of working back from the peak event/competition of the year when preparing their annual plan. However, they also noted that although there is often one main competition to peak for in a year, there are potentially multiple additional regattas that an athlete may also need to peak for (i.e., usually 3–4 international regattas per year). One coach specifically highlighted the prioritisation of periodising the annual training plan around the skills of the sport, rather than physiological fitness.

The third higher order theme, ‘volume of training’, refers to the combined frequency and duration of training sessions, and can be quantified over several time periods (e.g., week, month, year etc.) [66]. Coaches described both the volume of on-water training as well as the combined on- and off-water training:

*“Looking at that Olympic level, you’re on the water five days a week. Generally, you’re doing two and a half, three hours a day, on the water.” [P4]*

In comparison to other endurance sports, specific on-water ILCA training sessions are typically less frequent (~5 sessions per week), but longer in duration (2.5–3 h) [66]. With the addition of off-water training sessions such as cycling and resistance training, the overall training volume of ILCA sailors gets close to that of U23 cyclists (~750 h per year) but is still significantly lower than that of elite rowers (1100–1200 h per year) [67,68].

Furthermore, ‘mirroring regatta demands’ refers to matching the physical load of a worst-case (i.e., highest demand) regatta in training, to ensure athletes are physically prepared for the demands of competition. Coaches spoke to explicitly matching the regatta demands using several different variables (i.e., on-water and off-water volume and intensity), including non-peak competitions/regattas, in addition to the regatta demands being innately integrated within the training process:

*“I think we don’t train for the regatta specifically, you know, like we train to be fit and fast. The amount of hours that we spend makes the regatta feel easy. So it’s not a shock that you’re going to the regatta and suddenly, these type of feats... They should be so prepared that regatta shouldn’t be anything special.” [P9]*

This concept is not novel and has been investigated in team sports [69,70], where similarly to sailing, demands can change depending on opponent/s and environmental conditions. This highlights the importance of prescribing and monitoring an athlete’s training to ensure that they are appropriately prepared for the demands of a worst-case scenario regatta.

Additionally, ‘tapering’ has been defined as “a progressive nonlinear reduction of the training load during a variable period of time, in an attempt to reduce the physiological and psychological stress of daily training and optimise sports performance” [71] (p. 80). Most coaches noted the importance of ‘tapering’ before a competition so that the athlete is *“fresh”* to perform. In addition to the current practices of reducing training volume but maintaining training intensity, some coaches also highlighted the importance of having a chance to adapt to the conditions at the regatta venue:

*“But the final week usually would be at the venue, adapting to conditions. It’s more just a research week of what’s the wind? What are the wind tendencies? What are the water tendencies? Is there current? Land effects? And trying to put into place techniques that the sailor already has that are suited to the conditions at the venue.” [P3]*

Finally, the last higher order theme associated with periodisation was ‘keeping the joy’, with several coaches highlighting the need to make sure there was aspects of fun and enjoyment in training along the journey. It is widely known that an athlete’s adaptation to a given stimulus may not be predictable based on just a dose-response relationship, and background psycho-emotional influences play a key part in the adaptive response [72]. This has also been emphasised by a study in which coaches outlined the importance of non-physical factors (i.e., coach-athlete relationship, psychological and emotional stress, life stress, and an athletes’ belief in the plan) on influencing training adaptation [73]. This highlights the importance of ‘keeping the joy’ within the training program to improve an athlete’s ability to adapt to the imposed demands. Furthermore, coaches’ insights from this study align with Self Determination Theory (SDT) which identifies three psychological needs: autonomy, competence and relatedness [74,75]. To foster these, coaches could integrate SDT and principles of non-linear learning pedagogy, such as the use of modified interactive practice, questioning and cooperative learning to develop skill rather more rigid deliberate description and drilling of technique [74]. This approach enhances engagement and strengthens the coach-athlete relationship [74]. Additionally, implementing an iterative framework such as Action Research may help coaches to systematically plan, action and reflect on their ability to create an autonomy supportive learning environment which can enhance athlete adaptive motivation and improve their own coach development. Overall, ‘keeping the joy’ can be achieved by incorporating individualised, varied, and enjoyable training activities that foster a positive training environment.

Overall, the practical application of the higher order themes of ***periodisation*** such as ‘annual planning’, ‘mirroring regatta demands’, and ‘tapering’ help ILCA sailors, coaches and performance support staff optimise training programmes to peak at the right times for major competitions. Incorporating principles such as training volume management, ‘goal setting’, and ‘keeping the joy’ ensures that sailors not only prepare effectively but also maintain their enthusiasm and mental well-being throughout the rigorous demands of competitive ILCA sailing.

#### General dimension: *Specific Training.*

Training specificity centres around incorporating specific tasks of a sport to induce neuromuscular and metabolic adaptations to improve fitness/performance [76]. Four key higher order themes clustered together to establish the general dimension of ***specific training***: ‘hiking training’, ‘boat speed’, ‘off-water training’, and ‘training mental capabilities’ (Table 3).

The first higher order theme, ‘hiking training’, was an important factor that was discussed from both an on-water and off-water (i.e., hiking bench) perspective. The emphasis on this by the majority of coaches was to be expected given the importance placed on ‘hiking ability’ in the **determinants of athlete success,** in addition to the focus in the existing literature [7,31,34,50,77]. Coaches often felt that on-water hiking was the best way to get better at hiking, with the hiking bench used to supplement training when conditions or training logistics didn’t allow for on-water practice.

To corroborate this theme, regatta performance (Danish National Championship) has been observed to relate strongly with mean hiking strap force (809 ± 280 N, r = 0.79, p < 0.01), and the drop off (i.e., fatigue) in hiking strap force during the second bout of a 10 min upwind trial (r = 0.82, p < 0.05) in Europe (similar to an ILCA) sailing [53]. Additionally, recent research has shown that an eight-week hiking bench training program can delay the onset of fatigue during hiking by improving hiking economy and the activation of lower limb and trunk muscles [78]. Nonetheless, further research is warranted to explore the specifics of ‘hiking training’ (both on- and off-water) and its relationship with on-water hiking and regatta performance.

‘Boat speed’ was accentuated as a key focus in specific training, with coaches dedicating up to 80% of their training time to its improvement (Table 3). This emphasis aligns with coaches’ beliefs in the crucial role of boat speed for success in ILCA sailing (i.e., as previously expressed in **determinants of athlete success**, general dimension: ***sailing the boat fast***). The positive impact of ‘boat speed’ (often considered analogous to VMG) on performance is supported by studies in both competitive scenarios [10] and dinghy simulation [79], underscoring its importance in the training program.

All coaches described and highlighted aspects of ‘off-water training’ that were important to the overall training program and its specificity to different aspects of performance:

*“I think cycling, it is good for the specific performance, but it’s even better for the recovery performance, just endurance in general.” [P7]*

In addition to cycling, coaches also mentioned a range of other cross-training modalities such as rowing, swimming, rock climbing, water running, and resistance training. Strength and fitness sessions have been reported to be beneficial for increasing a sailor’s overall physical capabilities [2]. However, although off-water training is a crucial element to ensure athletes physical fitness does not compromise their performance, empirical research findings have not yet been shown a strong relationship between physical fitness/capabilities and regatta performance. Notably, this may be due to the contribution of a sailor’s skill and technical ability which accounts for a large (albeit difficult to quantify) part of racing performance [2].

Finally, coaches also mentioned ‘training the mental capabilities’ of their athletes. Due to the importance of cognitive skills highlighted in the **determinants of athlete success**, this may have been emphasised more, however, the interview guide and structure targeted areas more relevant to physical demands, which may have influenced the responses. Regardless, the importance of mental skills training in both sports performance and athlete well-being has been well established for over 40 years [39].

In conclusion, coaches and performance support staff can enhance ILCA sailing performance by designing targeted training programs that focus on the specific areas such as hiking technique, optimising boat speed, off-water training, and mental skills development.

### Limitations and future directions

Some limitations of this study require further consideration. Firstly, although **determinants of athlete success** were discussed, the interview was particularly focussed on the physical aspects of ILCA sailing, which may have detracted from other determinants of athlete success such as environmental, cultural, and socio-economic background etc. The current study was not set up to fully explore and examine these factors, and therefore, future research could investigate these variables of potential influence. Secondly, although mental abilities and skills were mentioned heavily in **determinants of athlete success**, the rest of the interview focussed more on the physical demands of training and competition, with coaches only making brief mention of mental aspects in these areas. Due to the highlighted importance of mental qualities in the determinants of athlete success, this is an area that should be explored in more depth in future research. Finally, as most coaches had primarily coached within Australia’s high performance sport system, perspectives from coaches from different countries and backgrounds may have provided other insights and nuanced views. Future research could explore this concept and delve into the experience and perceptions of a wider range of coaches from different countries and cultural backgrounds.

## Conclusion

The semi-structured interviews with experienced ILCA sailing coaches provided insights into the current landscape and thoughts within three key topic areas: i) **determinants of athlete success**, ii) **the physical demands of competition**, and iii) **training practices and philosophies**. Three general dimensions for **determinants of athlete success** were developed: i) ***sailing the boat fast***, ii) ***being a knowledgeable athlete***, and iii) ***consistent execution***. Within the **physical demands of competition**, three general dimensions were also established: i) ***hiking is the most physically demanding skill***, ii) ***environmental conditions influence athletic demands***, and iii) ***accumulation of fatigue over a regatta***. Finally, within the topic area of **training practices and philosophies**, there were two general dimensions: i) ***periodisation***, and ii) ***specific training***.

Notably, this study corroborates existing research findings, particularly around hiking, which consistently surfaced as a general dimension and higher order theme across *all three* topic areas. It is therefore evident that hiking should be considered as a crucial element within an ILCA sailor’s training program and future research could investigate the impact of different hiking training factors (e.g., duration, intensity, specificity, and frequency) on performance.

Beyond reinforcing established knowledge, our findings provide novel insights into key aspects of training and performance that have been scarcely researched in the sailing literature, particularly the concepts of ‘feel’ and ‘keeping the joy’. Developing ‘feel’ – the ability to perceive and respond to subtle changes in wind, water and boat movement – is widely recognised by coaches as critical to performance but is an underexplored skill in sailing. Future research should examine how ‘feel’ develops over time or how coaches can foster improvement through training methodologies and experiential learning. Similarly, ‘keeping the joy’ was identified as a crucial theme in maintaining athlete engagement and long-term development, which aligns with psychological frameworks (SDT) emphasising autonomy, competence and relatedness. Future research should examine psychological well-being strategies in high performance sailing programs to better utilise the benefits of addressing underlying psychological needs. Furthermore, although cognitive skills such as ‘pattern recognition’ and ‘decision making’ were not the primary focus of this study, our findings suggest that they play a significant role in ILCA sailing performance and clearly warrant further investigation.

By offering novel insights and actionable recommendations, this study provides athletes, coaches, and performance support practitioners with numerous considerations to refine training and physical preparation programs for ILCA sailors. These findings also contribute to a broader understanding of holistic athlete development within ILCA sailing and provide researchers with future directions and avenues of inquiry.
